# Denitrifying anaerobic methane-oxidizing bacteria in river networks of the Taihu Basin: Community dynamics and assembly process

**DOI:** 10.3389/fmicb.2022.1074316

**Published:** 2022-12-20

**Authors:** Ruyue Wang, Sai Xu, Yuxiang Zhu, Tao Zhang, Shijian Ge

**Affiliations:** ^1^Jiangsu Key Laboratory of Chemical Pollution Control and Resources Reuse, School of Environmental and Biological Engineering, Nanjing University of Science and Technology, Nanjing, China; ^2^Jiangsu Co-Innovation Center of Efficient Processing and Utilization of Forest Resources, College of Chemical Engineering, Nanjing Forestry University, Nanjing, China; ^3^Nanjing Institute of Environmental Sciences, Ministry of Ecology and Environment, Nanjing, China

**Keywords:** DAMO bacteria, spatiotemporal dynamics, river networks, community assembly, amplicon sequencing

## Abstract

Denitrifying anaerobic methane-oxidizing bacteria (DAMO bacteria) plays an important role in reducing methane emissions from river ecosystems. However, the assembly process of their communities underlying different hydrologic seasons remains unclarified. In this study, the dynamics of DAMO bacterial communities in river networks of the Taihu Basin were investigated by amplicon sequencing across wet, normal, and dry seasons followed by multiple statistical analyses. Phylogenetic analysis showed that Group B was the major subgroup of DAMO bacteria and significant dynamics for their communities were observed across different seasons (constrained principal coordinate analysis, *p* = 0.001). Furthermore, the neutral community model and normalized stochasticity ratio model were applied to reveal the underlying assembly process. Stochastic process and deterministic process dominated the assembly process in wet season and normal season, respectively and similar contributions of deterministic and stochastic processes were observed in dry season. Meanwhile, abundant (relative abundance >0.1%) and rare (relative abundance <0.01%) DAMO bacterial communities were found to be shaped *via* distinct assembly processes. Deterministic and stochastic processes played a considerable role in shaping abundant DAMO bacterial communities, while deterministic process mainly shaped rare DAMO bacterial communities. Results of this study revealed the dynamics of DAMO bacterial communities in river networks and provided a theoretical basis for further understanding of the assembly process.

## Introduction

Methane is the second most important greenhouse gas with a global warming potential 20–30 times that of carbon dioxide (Malyan et al., 2016). In recent decades, atmospheric methane concentrations have been increasing at an annual rate of 1.0–1.2%, attracting widespread attentions (Borrel et al., 2011; Malyan et al., 2016).

Microbial-mediated methane oxidation is an effective pathway to reduce methane emissions (Malyan et al., 2016; Wang et al., 2019; Xu and Zhang, 2022). According to the availability of oxygen, methane oxidation can be divided into two distinct processes: aerobic oxidation of methane and anaerobic oxidation of methane (AOM). In freshwater ecosystem, denitrifying anaerobic methane oxidation (DAMO) is the most widely investigated AOM pathway (Shen et al., 2014; Cheng et al., 2022), which can couple methane oxidation with denitrification by using nitrate/nitrite as the electron acceptors (Strous and Jetten, 2004; Raghoebarsing et al., 2006). DAMO bacteria, belonging to the NC10 phylum (Ettwig et al., 2010), is one of the major functional microorganisms participating in DAMO process. Up to now, five phylogenetically distinct subgroups of DAMO bacteria (Groups A–E) have been verified (Chen et al., 2016), among which Group A and Group B were widely distributed in freshwater ecosystem. For example, Group B was more abundant than Group A in the Qiantang River (Shen et al., 2014), while the dominant subgroups were found to be distinct in different reaches in the Wuxijiang River (Cheng et al., 2022). However, the dynamics of different subgroups of DAMO bacteria and the underlying driving factors remained largely unknown.

Revealing the assembly process that shape the microbial community is one of the central topics in microbial ecology of freshwater ecosystems (Logares et al., 2018; Sjostedt et al., 2018). Deterministic and stochastic processes are two main mechanisms that shape the microbial communities (Zhou and Ning, 2017). Deterministic processes refer to that environmental factors and species interactions are the main determinants in shaping microbial communities (Nemergut et al., 2013; Vanwonterghem et al., 2014). In contrast, stochastic processes believe that random factors, such as birth, death, speciation, limited dispersal, and immigration, shape microbial communities (Chave, 2004; Zhou and Ning, 2017). Previous studies have confirmed the distribution of DAMO bacteria in freshwater ecosystems with focus on its diversity and abundance (Shen et al., 2014; Cheng et al., 2022), while the underlying community assembly process is still unclear. Moreover, microbial communities often exhibit an uneven distribution, with a few abundant taxa coexisting with a large number of rare taxa (Fuhrman, 2009; Jia et al., 2018). Recently, several studies have reported that the abundant and rare bacterial communities exhibited distinct assembly processes (Hou et al., 2020; Yang et al., 2022), and whether this applies to DAMO bacterial communities remains unknown.

Rivers are typical freshwater ecosystems and serve as key transport channels for the global carbon and nitrogen biogeochemical cycles (Grill et al., 2019; Maavara et al., 2020). In recent decades, rivers in the Taihu Basin have been seriously polluted by the excessive load of nitrogen, providing a large amount of potential electron acceptors for DAMO process (Ju et al., 2016; Wu et al., 2022), and thus, it is an ideal habitat for DAMO bacteria. The Taihu Basin lies in subtropical monsoon climate zone and rivers located in this region experience remarkable wet-normal-dry cycles each year. Previous studies showed that the environmental conditions induced by the wet-normal-dry cycles could affect the microeukaryotic and bacterioplankton communities (Gad et al., 2020; Zhang et al., 2022). Hence, it is reasonable to speculate the dynamics of DAMO bacterial communities across different hydrologic seasons. In this study, amplicon sequencing was employed to analyze the diversity and assembly process of DAMO bacterial communities across three hydrologic seasons (wet, normal, and dry seasons). This study aimed to (1) explore the spatiotemporal dynamics of DAMO bacterial communities across different hydrologic seasons; (2) determine the relative roles of deterministic and stochastic processes in assembly of DAMO bacterial communities; and (3) identify major environmental drivers in shaping the DAMO bacterial communities. Results of this study will be helpful in understanding the dynamics and assembly process of DAMO bacterial communities in the freshwater ecosystem under hydrological disturbance.

## Materials and methods

### Study area and sample collection

The Taihu Basin is located in the Yangtze River Delta in eastern China, covering an area of 36,900 km^2^, with the water area constituting 17% of the total area. Sampling campaign was carried out at 18 sampling sites on four crisscross rivers (Beijing-Hangzhou Grand Canal, Wujin -Yixing Canal, Danjinlicao River, and Yili River; Supplementary Figure S1) during July 2020 (wet season), October 2020 (normal season), and January 2021 (dry season). Due to some unforeseen reasons, we failed to collect samples from one site in normal season and three sites in dry seasons (Supplementary Table S1). At each sampling site, the upper layer (~10 cm) of the sediments was collected using the grab bucket. The sediment samples were put into sterile plastic bags and transported to the laboratory within 4 h under low temperature (4°C). Each sample was immediately divided into two parts: One part was stored at 4°C for physicochemical analyses and the other part was stored at −20°C for microbial analyses.

### PCR amplification, amplicon sequencing, and data analyses

~0.5 g samples were used for microbial DNA extraction by FastDNA® SPIN Kit for Soil (MP biomedicals, United States). The nested PCR amplification method was used to amplify the 16S rRNA gene of DAMO bacteria, consisting of an initial PCR with primers qP1mF (5′-GGGCTTGAC ATCCCACGAACCTR-3′)/1492R (5′-ACGGCTACCTTGTTACGACTT-3′; Kane et al., 1993) followed by the second round of PCR with primers 1051F (5′-ARCGTGGAGACAGGTGGT-3′)/qP2R (5′-CTCAGCGACTTCGAGTACAG-3′; He et al., 2016b). The PCR products were sequenced on Illumina’s NovaSeq 6000 platform at the Novogene Company (Tianjin, China). All of the raw sequencing reads have been deposited in the Genome Sequence Archive in National Genomics Data Center^1^ with accession number CRA005757.

The sequencing data were analyzed by the DADA2 pipeline following the tutorial (Callahan et al., 2016). Briefly, the raw sequence reads were firstly split into different samples by matching the barcodes. The barcodes, adaptors, and primers were then filtered using the command “filterAndTrim.” The sequences were duplicated and denoised using the “duplicated” and “dada” commands, respectively. The pair-end sequence reads were merged using the “mergePairs” command, and the chimeras were removed using the “removeBimeraDenovo” command. Finally, the amplified sequence variant (ASV) table was generated using the “makeSequenceTable” command. The representative sequence from each ASV was blasted with four DAMO bacterial genomes in the GenBank database, including *Candidatus Methylomirabilis oxyfera* (*M. oxyfera*, NR_102979.1; Ettwig et al., 2010), *Candidatus Methylomirabilis sinica* (*M. sinica*, KU891931.1; He et al., 2016a), *Candidatus Methylomirabilis limnetica* (*M. limnetica*, NVQC01000015.1; Graf et al., 2018), and *Candidatus Methylomirabilis lanthanidiphyla* (*M. lanthanidiphyla*, CABIKM010000010.1; Versantvoort et al., 2018). The ASVs with similarity >85% to any of *M. oxyfera*, *M. sinica*, *M. limnetica*, and *M. lanthanidiphyla* were remained for downstream analyses. The abundant and rare ASVs were defined following a previous study (Cui et al., 2021). ASVs with relative abundances >0.1% were defined as “abundant” DAMO bacterial communities, and those with relative abundances <0.01% were defined as “rare” DAMO bacterial communities.

The alpha diversity (Shannon index and Observed richness), and beta diversity index (“Bray-Curtis” distance) were calculated by “vegan” package (version 2.6-2) in the R software (version 4.0.2). One-way ANOVA followed by Duncan’s multiple comparison test was used to compare the alpha diversity and beta diversity across different seasons by “agricolae” package (version 1.3-3) in the R software (version 4.0.2).

The variations of DAMO bacterial communities across different hydrologic seasons were revealed *via* constrained principal coordinate analysis (CPCoA) and permutation multivariate ANOVA (PERMANOVA). The CPCoA and PERMANOVA were performed by “amplicon” package (version 1.1.4) and “vegan” package (version 2.6-2) in the R software (version 4.0.2), respectively. The distance-decay patterns of DAMO bacterial communities were calculated as the slope of the linear regression between geographic distance and “Bray-Curtis” similarity (1- “Bray-Curtis” distance) of each pairwise sample. The habitat niche breadth (*Bcom*) of each ASV was calculated by “spaa” package (version 0.2.2) in the R software (version 4.0.2; Zhou et al., 2014). The differences in *Bcom* values were analyzed using One-way ANOVA followed by Duncan’s multiple comparison test.

Typical ASVs (top 10 most abundant) from DAMO bacterial communities were selected for phylogenetic analysis. Phylogenetic analysis was performed by MEGA 7 software using the neighbor-joining method with 1000 bootstrap replicates (Kumar et al., 2016).

### Physicochemical analyses

Ammonia nitrogen (NH_4_^+^-N), nitrite nitrogen (NO_2_^−^-N), and nitrate nitrogen (NO_3_^−^-N) were extracted with 2 mol/L KCl solution for 1 h and then measured colorimetrically by a spectrophotometer (Shimadzu, Japan; Long et al., 2017; Xu et al., 2017a). Dissolved organic carbon (DOC) was extracted by ultra-pure water and quantified using a TOC analyzer (Elementar, Germany). Metals (Cd, Cr, Cu, Mn, Ni, and Zn) were firstly digested by combined acids (HNO_3_-HClO_4_-HF) and then determined by the ICP-OES (PerkinElmer, United States; Xu et al., 2017b). The pH was measured by a pH meter (Mettler Toledo, Switzerland) with a solid to ultra-pure water ratio of 1:2.5 (Shi et al., 2021). The physicochemical attributes were listed in Supplementary Table S2.

The distance-based (Bray-Curtis distance) redundancy analysis (db-RDA) and Mantel test were carried out to reveal the relationships between DAMO bacterial communities and environmental factors. The db-RDA and Mantel test were conducted using “vegan” package (version 2.6-2) and “linkET” package (version 0.0.3.3) in the R software (version 4.0.2), respectively.

### Community assembly analyses

The neutral community model (NCM) was used to evaluate the potential contribution of stochastic process in community assembly by predicting the relationship between detection frequency and relative abundance of each ASV (Sloan et al., 2006). In this model, positive R^2^ represented the fit of the entire neutral model. The 95% confidence intervals were calculated based on 1,000 bootstrap replicates.

The normalized stochasticity (NST) ratio was further applied to evaluate the relative importance of deterministic and stochastic processes in shaping DAMO bacterial communities (Ning et al., 2019). This model used 50% as the boundary point between deterministic (NST < 50%) and stochastic (NST > 50%) assembly processes. The NST values based on “Bray-Curtis” similarity were calculated by “NST” package (version 3.1.9) in the R software (version 4.0.2).

## Results

### Phylogenetic analysis of DAMO bacterial communities

In this study, a total of 2,537,849 high-quality sequences were obtained by amplicon sequencing and they were classified into 2,714 ASVs. ASVs with less than 85% identity to *M. oxyfera*, *M. sinica*, *M. limnetica*, and *M. lanthanidiphyla* were filtered and 2,599 ASVs (2,535,087 sequences) were obtained. After removing ASVs with less than 5 sequences to reduce the PCR bias (Naylor et al., 2017), a total of 2,044 ASVs (2,533,420 sequences) were retained, ranging from 38,796 to 78,242 sequences per sample. Each sample was finally rarefied to the minimum sequences (38,796) for downstream analyses.

**Figure 1:**
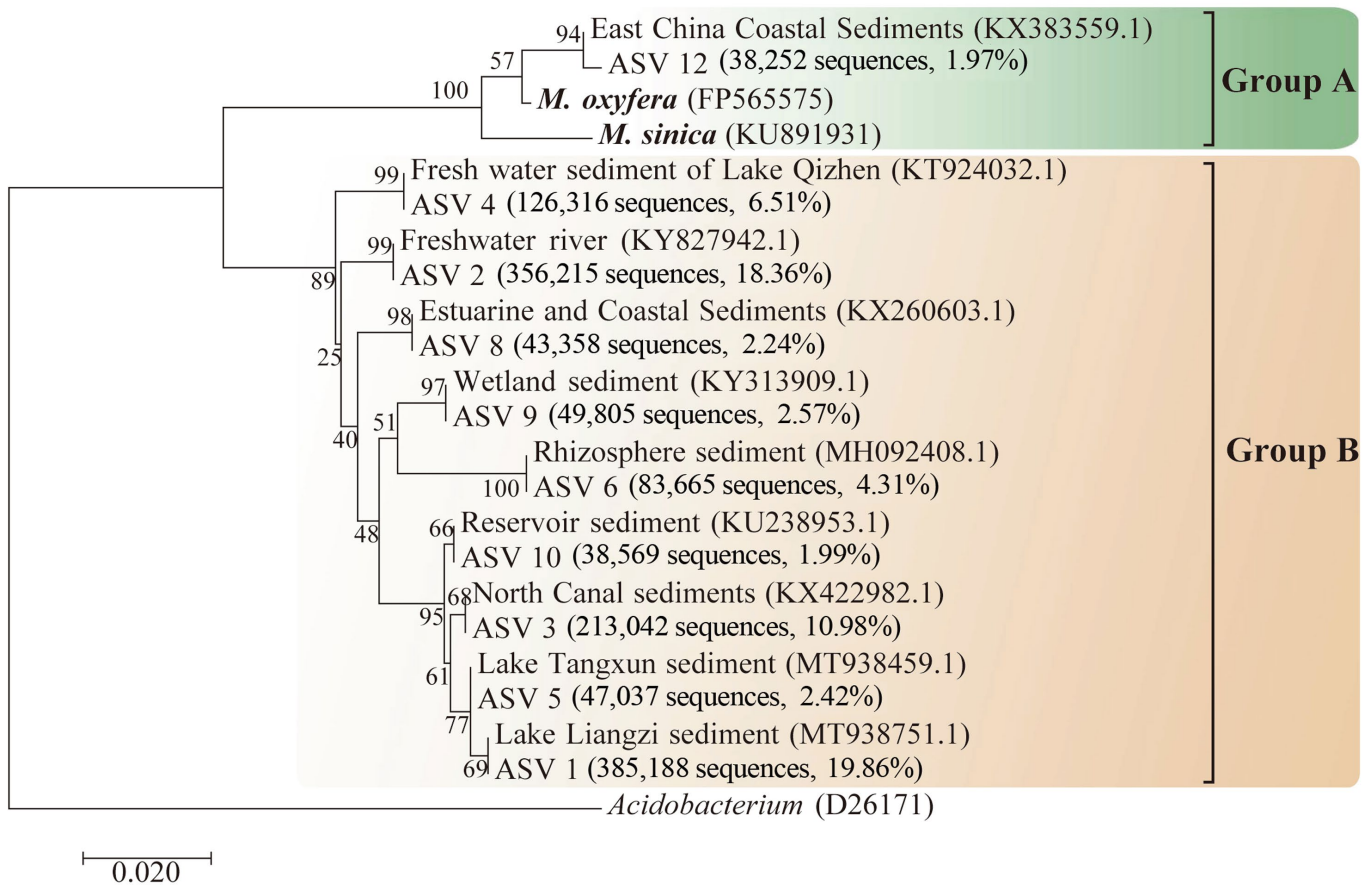
Neighbor-joining phylogenetic tree showing the phylogenetic affiliations of denitrifying anaerobic methane oxidation (DAMO) bacteria. Bootstrap values were calculated by 1,000 replicates, and the scale bar represented 2% sequence divergence.

Typical ASVs (top 10 most abundant, Supplementary Table S3) from DAMO bacterial communities were selected for phylogenetic analysis (Figure 1). These ASVs accounted for 71.22% of total sequences and they were divided into two subgroups: Group A and Group B (Ettwig et al., 2009). The phylogenetic analysis showed that one ASV (ASV12) was grouped into Group A, with 98.39 and 97.58% identity to *M. oxyfera* and *M. sinica*, respectively. This ASV was more abundant in normal season (63.80%) than the other seasons (Supplementary Table S4). The other ASVs were clarified as Group B (Figure 1) and the proportions of Group B were almost the same during wet, normal, and dry seasons (Supplementary Table S4). These results showed that the Group B was the dominant subgroup and subgroups of DAMO bacterial communities exhibited seasonal dynamics.

### Spatiotemporal dynamics of DAMO bacterial communities

**Figure 2:**
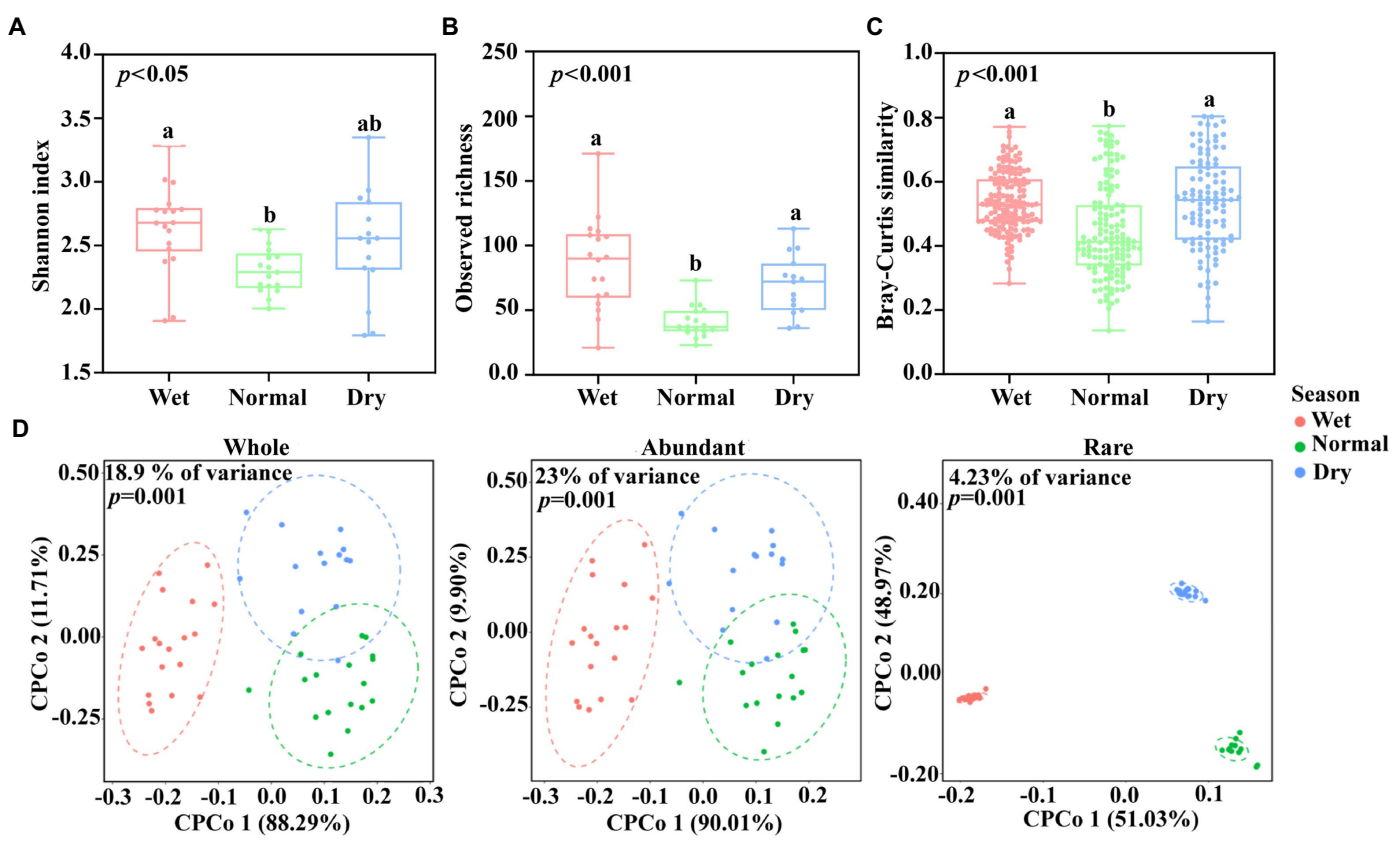
Dynamics of **(A)** Shannon index, **(B)** Observed richness, and **(C)** Bray–Curtis similarity of DAMO bacterial communities during wet, normal, and dry seasons. Different letters indicated significant difference by Duncan test (*p* < 0.05). **(D)** Constrained principal coordinate analysis (CPCoA) plot based on Bray-Curtis distance showing the variation of DAMO bacterial communities.

Significant temporal dynamics of alpha diversity and beta diversity of DAMO bacterial communities were observed across different seasons (Figures 2A–C). The Shannon diversity index was found to be higher in wet season than normal season (Figure 2A, *p* < 0.05) and wet and dry seasons showed higher richness than normal season (Figure 2B, *p* < 0.001). Meanwhile, the similarity of DAMO bacterial communities also exhibited temporal dynamics (Figure 2C), and the community similarity during wet and dry seasons was significantly higher than that of normal season (*p* < 0.001).

To compare the profile of abundant and rare DAMO bacterial communities, 48 ASVs with 1,688,209 (66.59%) sequences and 1,662 ASVs with 55,185 (2.18%) sequences were selected as the “abundant” and “rare” DAMO bacterial communities, respectively. The CPCoA plot and PERMANOVA showed clear differences in whole, abundant, and rare DAMO bacterial communities during wet, normal, and dry seasons (Figure 2D; Supplementary Table S5). These results indicated that the whole, abundant, and rare DAMO bacterial communities exhibited similar temporal dynamics. The Venn diagram showed that the shared ASVs in abundant DAMO bacterial communities (77.1%) were far more than those in whole (3.77%) and rare (0.18%) DAMO bacterial communities across different seasons. Up to 97.7% ASVs in rare DAMO bacterial communities were found to be unique across different seasons (Supplementary Figure S2). This result implied that biodiversity patterns differed between abundant and rare DAMO bacterial communities.

The spatial dynamics of DAMO bacterial communities were revealed by distance-decay patterns. The dissimilarity of DAMO bacterial communities increased with geographical distance during normal and dry seasons (Supplementary Figure S3). Similarly, the abundant DAMO bacterial communities also exhibited distance-decay patterns during normal and dry seasons (*p* < 0.001). On the contrary, the rare DAMO bacterial communities did not show significant distance-decay patterns (Supplementary Figure S3). These results indicated that abundant and rare bacterial communities exhibited dissimilar spatial patterns across different seasons.

### Assembly process of DAMO bacterial communities

**Figure 3:**
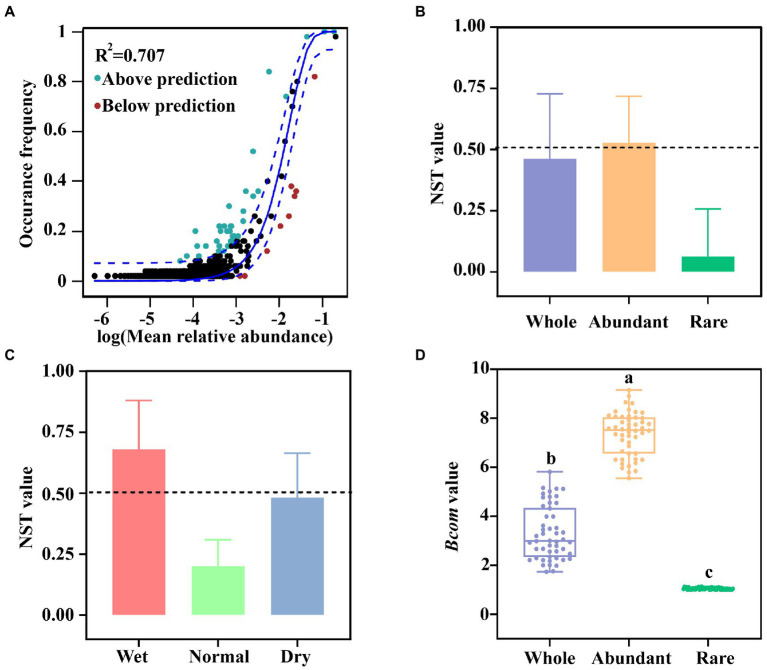
The assembly process and habitat niche breadth for whole, abundant, and rare DAMO bacterial communities. **(A)** Fit of the NCM model. The solid lines indicated the best fit to the NCM model, and the dashed lines represented 95% confidence intervals around the model prediction. ASVs that occurred more or less frequently than predicted by the NCM model were shown in green and red colors, respectively. *R*^2^ indicated the fit to this model. **(B)** The assembly process for whole, abundant, and rare DAMO bacterial communities based on NST values. **(C)** The assembly process of DAMO bacterial communities during wet, normal, and dry seasons. **(D)** Boxplot showing *Bcom* values for whole, abundant, and rare DAMO bacterial communities. Different letters indicated significant difference (*p* < 0.001, Duncan test).

The NCM model was applied to evaluate the contribution of the stochastic process in assembly of DAMO bacterial communities (Figure 3A). The NCM model explained 70.7% (*R*^2^ = 0.707) for variations of DAMO bacterial communities and fitted well for the DAMO bacterial communities. Similar observation was found during wet, normal, and dry seasons (Supplementary Figure S4). These results together indicated that stochastic process shaped the DAMO bacterial communities to some extent.

The NST model was employed to further quantify the importance of deterministic and stochastic processes in shaping DAMO bacterial communities (Figure 3B). The mean NST value of DAMO bacterial communities was 46.17%, indicating that both deterministic and stochastic processes played a considerable role in shaping DAMO bacterial communities (Figure 3B). During different seasons, the assembly processes were found to be distinct. The stochastic process dominated the assembly process in wet season, while the deterministic process was the major process in shaping the DAMO bacterial communities in normal season. Similar contributions of deterministic and stochastic processes were observed in dry season (Figure 3C).

Similar to the whole DAMO bacterial communities, both deterministic and stochastic processes played important roles in shaping abundant DAMO bacterial communities (Figure 3B). During wet and dry seasons, the relative contribution of stochastic process was slightly greater than that of deterministic process (Supplementary Figure S5). Meanwhile, the deterministic process was found to be the dominant process in shaping rare DAMO bacterial communities during all of the three seasons (Supplementary Figure S5). Further, the average *Bcom* value of rare DAMO bacterial communities were significantly lower than that of the whole and abundant DAMO bacterial communities (Figure 3D, *p* < 0.001), which indicated that rare DAMO bacterial communities had narrower niche widths.

### Relationships between DAMO bacterial communities and environmental factors

**Figure 4:**
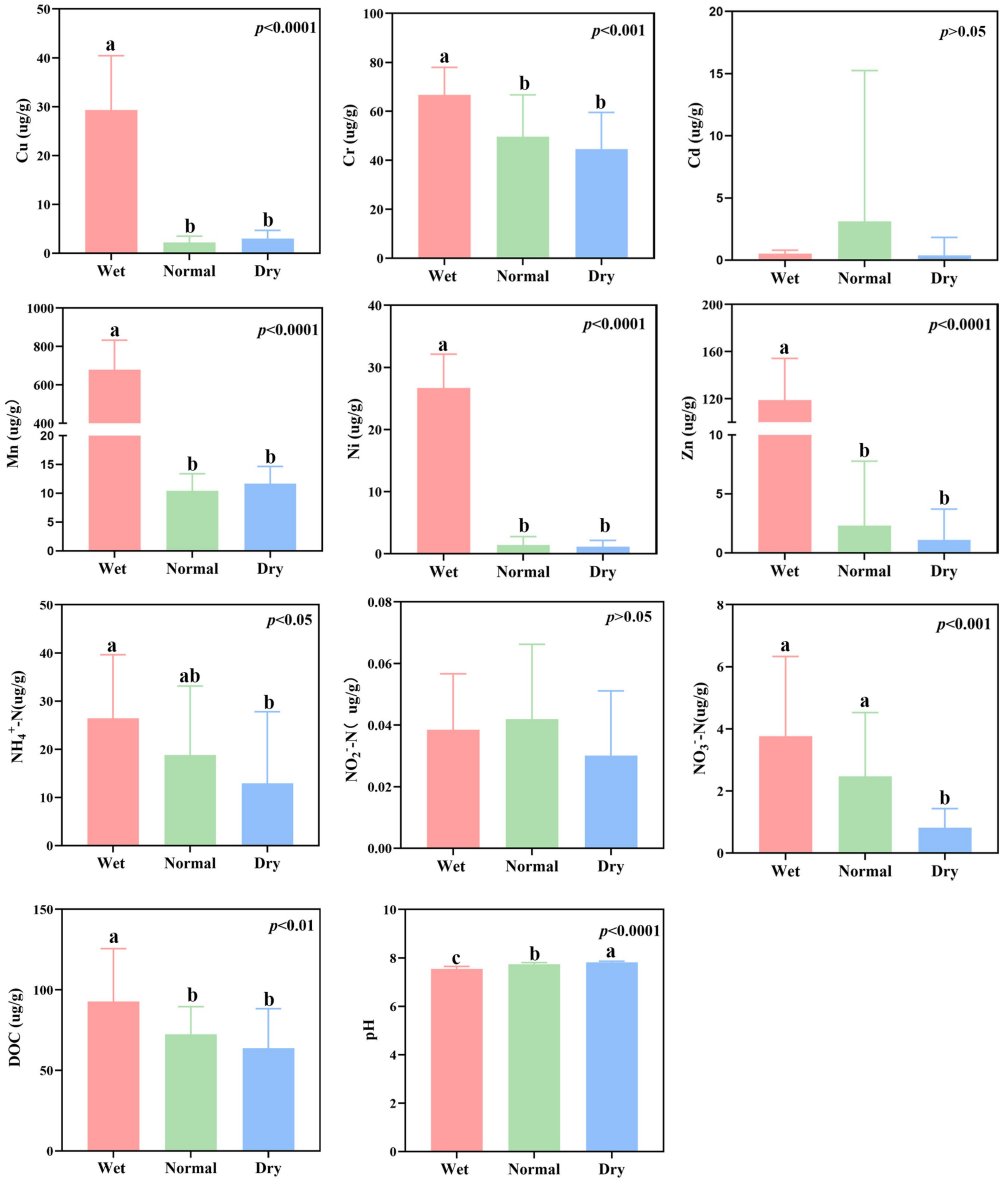
One-way ANOVA showing the dynamics of environmental factors during wet, normal, and dry seasons. Different letters indicated significant difference by Duncan test (*p* < 0.05).

**Figure 5:**
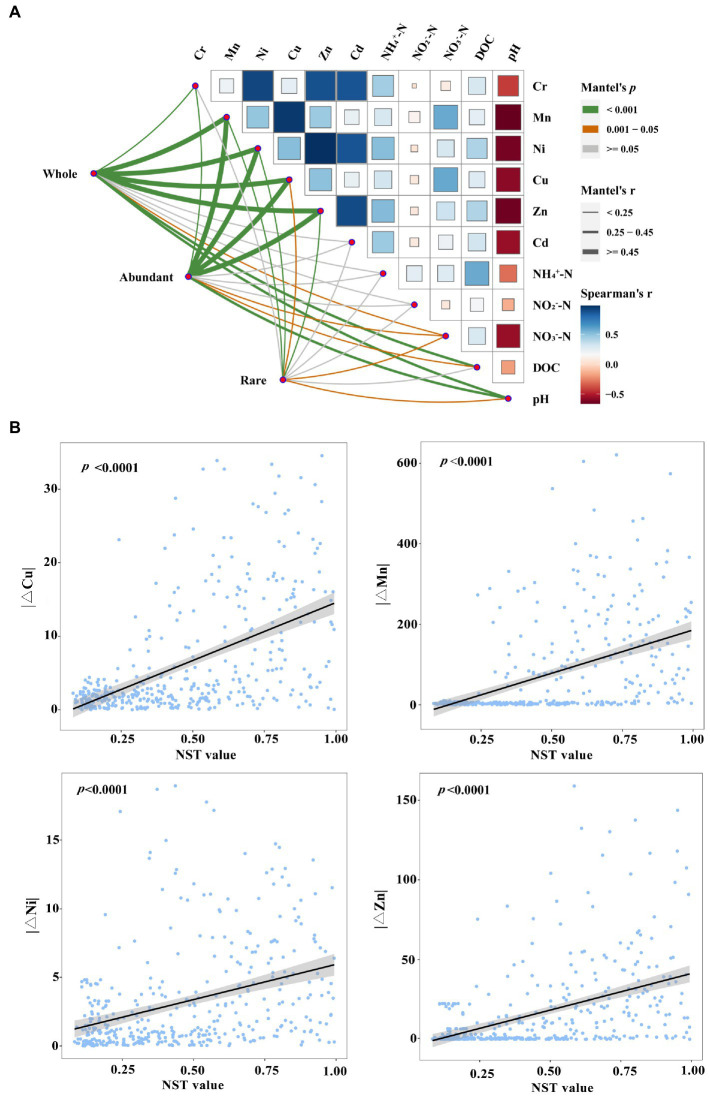
The environmental drivers DAMO bacterial communities. **(A)** Environmental drivers of the whole, abundant, and rare DAMO bacterial communities evaluated by Mantel test. Edge width corresponded to correlation coefficient and edge color indicated statistical significance. Pairwise comparisons of environmental variables with a color gradient represented Spearman correlation coefficients. **(B)** The linear relationship between NST values and metals (Cu, Mn, Ni, and Zn).

In this study, most of the environmental factors, such as Cu, Cr, DOC, Mn, Ni, NH_4_^+^-N, NO_3_^−^-N, pH, and Zn, exhibited significant temporal dynamics across different seasons (Figure 4, *p* < 0.05; Supplementary Table S2). The Mantel test revealed significant correlations between DAMO bacterial communities and metals (Cu, Mn, Ni, and Zn; Figure 5A; *r* > 0.45, *p* < 0.001). The db-RDA was further used to reveal the impacts of environmental factors on DAMO bacterial communities (Supplementary Figure S6). Based on the Monte Carlo test with 999 permutations, the db-RDA conformed that metals (Cu, Mn, Ni, and Zn) exhibited significant impacts on DAMO bacterial communities (*p* < 0.001). In addition, the linear regression analysis showed that these metals were significantly correlated with the NST values (Figure 5B, *p* < 0.0001). These results indicated that metals (Cu, Mn, Ni, and Zn) were the major driving factors affecting the structure and assembly process of DAMO bacterial communities. Meanwhile, similar to the whole DAMO bacterial communities, these metals showed strong correlations with abundant DAMO bacterial communities (Figure 5A; *r* > 0.45) while their correlations with the rare DAMO bacterial communities were weak (Figure 5A; *r* < 0.25), indicating that abundant and rare DAMO bacterial communities responded differently to environmental factors.

## Discussion

### Dynamics of DAMO bacterial communities

Previous studies have confirmed the distribution of DAMO bacteria in the river ecosystem (Shen et al., 2014; Cheng et al., 2022), while the dynamics of different subgroups of DAMO bacteria remain poorly explored. In this study, amplicon sequencing combined with phylogenetic analysis was applied to track the dynamics of DAMO bacterial communities in the river ecosystem. Our results showed that DAMO bacteria were mainly divided into Group A and Group B with Group B as the dominant subgroups (Figure 1). This observation was consistent with previous studies in other rivers (Shen et al., 2014; Cheng et al., 2022), indicating that Group B might be more adaptable in river ecosystems than Group A. Previous studies have confirmed that Group A had the ability to oxidize methane by enrichment culture and genomic analysis (Ettwig et al., 2009; He et al., 2015; Wang et al., 2016; Xu et al., 2018). Recent studies have also enriched Group B, indicating that it may also have the ability to oxidize methane (Hatamoto et al., 2017; Ma et al., 2017). These results implied that the DAMO process may play an important role in reducing methane emissions in the river ecosystem, while its *in-situ* activity needs to be further explored.

In this study, the diversity of DAMO bacterial communities was observed to exhibit temporal dynamics (Figures 2A–C). The alpha diversity (Shannon index) of DAMO bacterial communities was relatively more diverse in wet season. In wet season, rainfall may bring microorganisms from the surrounding system into the river, making DAMO bacterial communities more diverse. This was in line with a study exploring the dynamics of microeukaryotic community in Tingjiang River. Meanwhile, the structure of DAMO bacterial communities was also observed to exhibit temporal changes (Figure 2D; Supplementary Table S5). Several reasons may explain these results. Firstly, different hydrological conditions could lead to different environmental factors across three seasons, which can induce the variations of bacterial community diversity and structure (Figures 4, 5A; Supplementary Figure S6). Previous studies have confirmed this by showing that river flow, hydrological connectivity, and hydraulic retention time can modulate microbial community diversity and structure (Isabwe et al., 2018; Chen W. et al., 2019; Gad et al., 2020). Besides, the four crisscross rivers were interconnected and tended to have similar environmental condition in the same season, possibly leading to the seasonal clusters of DAMO bacterial communities.

The distance-decay patterns were widely prevalent in microbial geographic distributions (Hanson et al., 2012; Wang et al., 2017). In this study, the distance-decay patterns were only observed in normal and dry seasons, but not in wet season (Supplementary Figure S3), which was consistent with a previous study (Wang et al., 2015). This was reasonable since higher river flow improved hydrological connectivity and increased dispersal event, leading to higher community similarity in wet season.

### Assembly process of DAMO bacterial communities

Revealing the assembly process was important for understanding the microbial communities (Isabwe et al., 2018; Liu et al., 2022). The NCM and NST models were used to evaluate the assembly process of DAMO bacterial communities and the results showed that deterministic and stochastic processes played similar roles in assembly process of DAMO bacterial communities (Figures 3A,B). Similar observation was reported for the assembly process of bacterioplankton community (Nyirabuhoro et al., 2020). Further, the linear regression analysis, Mantel test, and db-RDA together revealed that metals were the major drivers to affect the structure and assembly process of DAMO bacterial communities (Figures 5A,B; Supplementary Figure S6). Previous studies have confirmed that the metal elements could affect the community and activity of DAMO bacteria (Unal et al., 2012; Wang et al., 2022), which may further affect the community assembly process.

The respective roles of deterministic process and stochastic process in shaping DAMO bacterial communities were found to be distinct in different seasons. The stochastic process dominated the community assembly process in wet season, while deterministic process was the major process in normal season (Figure 3C). In the wet season, frequent hydrological exchanges increased dispersal events (stochastic process) for DAMO bacterial communities. In contrast, in normal season, passive dispersal was not strong due to weaker hydrological connectivity and lower river flows. This was supported by previous studies that this period acted as a harsh environmental filter due to niche selection (Dudgeon et al., 2006; Chase, 2007), leading to deterministic process dominating community assembly process. This result was also consistent with distance-decay patterns that were not observed in wet season (Supplementary Figure S3).

Abundant and rare DAMO bacterial communities were observed to exhibit distinct assembly processes. Similar to the whole DAMO bacterial communities, the abundant DAMO bacterial communities were dominated by both deterministic and stochastic processes, while deterministic process shaped the rare DAMO bacterial communities (Figure 3B). Our results were consistent with previous studies on community assembly for abundant and rare taxa (Wan et al., 2021; Zhu et al., 2021). Previous studies have shown that taxa with narrow niche widths were more susceptible to deterministic process (Pandit et al., 2009; Xu et al., 2021; Zhou and Wu, 2021). In this study, rare DAMO bacterial communities had significant narrower niche widths than abundant DAMO bacterial communities (Figure 3D), confirming that deterministic process shaped the rare DAMO bacterial communities. Meanwhile, the distance-decay patterns of abundant DAMO bacterial communities were observed in normal and dry seasons while the rare DAMO bacterial communities did not exhibit a distance-decay pattern in all of the three seasons (Supplementary Figure S3). This result indicated that the abundant and rare DAMO bacterial communities may respond differently to changes in environmental factors. The Mantel test confirmed this by showing that abundant and rare DAMO bacterial communities exhibited different environmental sensitivities (Figure 5A), which could further affect their assembly processes.

## Conclusion

This study explored the dynamics and assembly process of DAMO bacterial communities in river networks of the Taihu Basin across different hydrologic seasons. Our results revealed that the diversity and structure of DAMO bacterial communities exhibited significant temporal dynamics while the spatial dynamics were found to be distinct in different seasons. The NCM and NST models together showed that stochastic and deterministic processes both played a considerable role in shaping DAMO bacterial communities. In addition, the abundant DAMO bacterial communities exhibited similar spatiotemporal variations, assembly process, and environmental response with the whole DAMO bacterial communities, but distinct from the rare DAMO bacterial communities. These results provided new clues for DAMO bacterial communities while their contributions to methane reduction need further investigation.

## Data availability statement

The datasets presented in this study can be found in online repositories. The names of the repository/repositories and accession number(s) can be found in the article/Supplementary material.

## Author contributions

SX and SG provided initial concept. RW and TZ carried out field sampling and chemical analyses. RW, SX, YZ, and SG wrote the manuscript. All authors contributed to the manuscript and approved the submitted version.

## Funding

This study was supported by National Natural Science Foundation of China (No. 42007302), Natural Science Foundation of Jiangsu Province (No. BK20190481), China Postdoctoral Science Foundation (No. 2020M681480), and Fundamental Research Funds for the Central Universities (No. 30922010810).

## Conflict of interest

The authors declare that the research was conducted in the absence of any commercial or financial relationships that could be construed as a potential conflict of interest.

## Publisher’s note

All claims expressed in this article are solely those of the authors and do not necessarily represent those of their affiliated organizations, or those of the publisher, the editors and the reviewers. Any product that may be evaluated in this article, or claim that may be made by its manufacturer, is not guaranteed or endorsed by the publisher.

## Supplementary material

The Supplementary material for this article can be found online at: https://www.frontiersin.org/articles/10.3389/fmicb.2022.1074316/full#supplementary-material

## Footnotes

^1^ https://ngdc.cncb.ac.cn/gsa
