# Supplementary material for: Denitrifying anaerobic methane-oxidizing bacteria in river networks of the Taihu Basin: Community dynamics and assembly process
Source: Front Microbiol. 2022 Dec 20;13:1074316. doi: 10.3389/fmicb.2022.1074316 (PMC9808034; doi:10.3389/fmicb.2022.1074316)
Supplement: Supplementary file 1 [file Data_Sheet_1.pdf]

Supplementary materials

Denitrifying anaerobic methane-oxidizing bacteria in river networks of the Taihu Basin: Community dynamics and assembly process

Ruyue Wang[a,1], Sai Xu[a,1,*], Yuxiang Zhu[b], Tao Zhang[c], Shijian Ge[a,*]

[a] Jiangsu Key Laboratory of Chemical Pollution Control and Resources Reuse, School of Environmental and Biological Engineering, Nanjing University of Science and Technology, Nanjing 210094, China

[b] Jiangsu Co-Innovation Center of Efficient Processing and Utilization of Forest Resources, College of Chemical Engineering, Nanjing Forestry University, Nanjing 210037, China

[c] Nanjing Institute of Environmental Sciences, Ministry of Ecology and Environment, Nanjing 210042, China.

[1] These two authors have contributed equally to this work and share first authorship.

* Corresponding author. E-mail address: xusai@njust.edu.cn (Sai Xu); geshijian1221@njust.edu.cn (Shijian Ge).

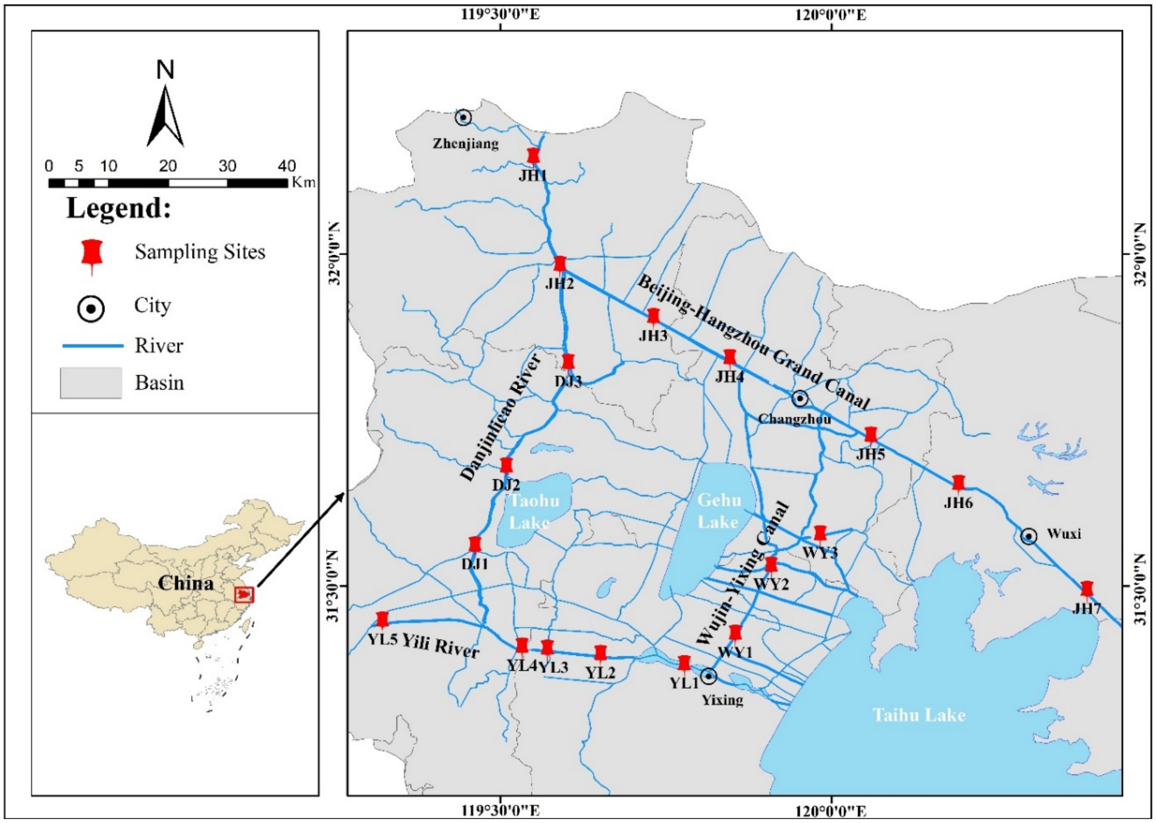

Figure S1 Map of sampling sites. Beijing-Hangzhou Grand Canal (JH1-JH7); Wujin - Yixing Canal (WY1-WY3); Danjinlicao River (DJ1-DJ3); Yili River (YL1-YL5).

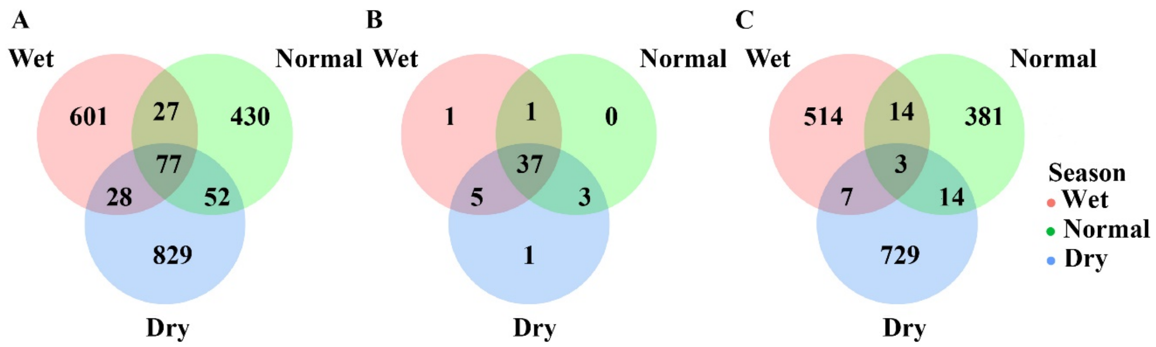

Figure S2 Venn diagram showing the shared and unique ASVs of (A) whole, (B) abundant, and (C) rare DAMO bacterial communities across different seasons.

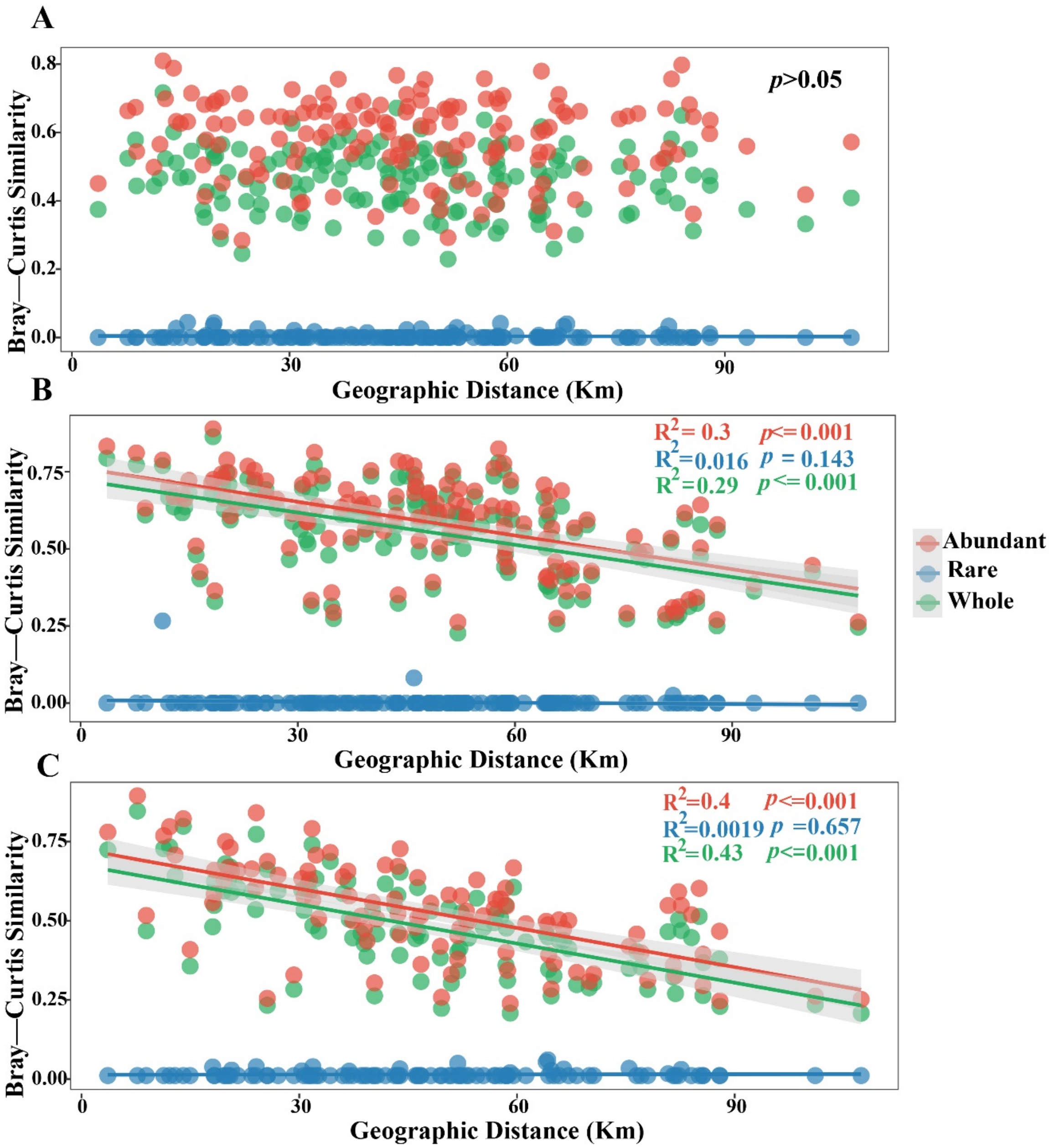

Figure S3 Distance-decay patterns based on the Bray-Curtis similarity (1-"Bray-Curtis" distance) of DAMO bacterial communities and geographic distance during (A) wet, (B) normal, and (C) dry seasons.

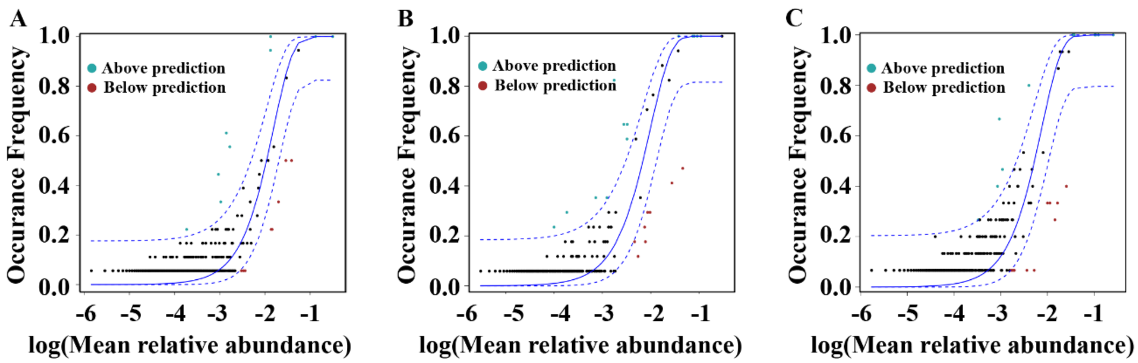

Figure S4 Fit of DAMO bacterial communities during (A) wet, (B) normal, and (C) dry seasons with NCM model.

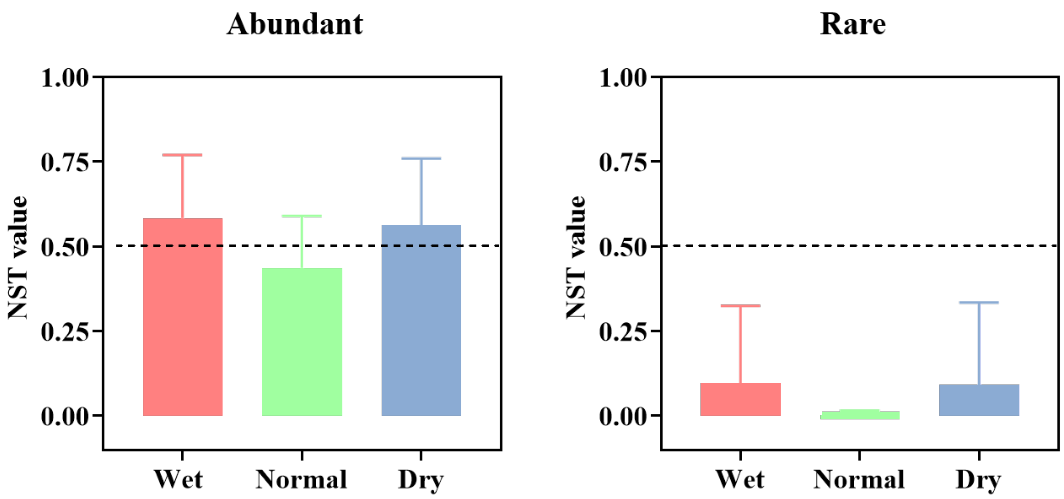

Figure S5 The assembly process of abundant and rare DAMO bacterial communities during wet, normal, and dry seasons.

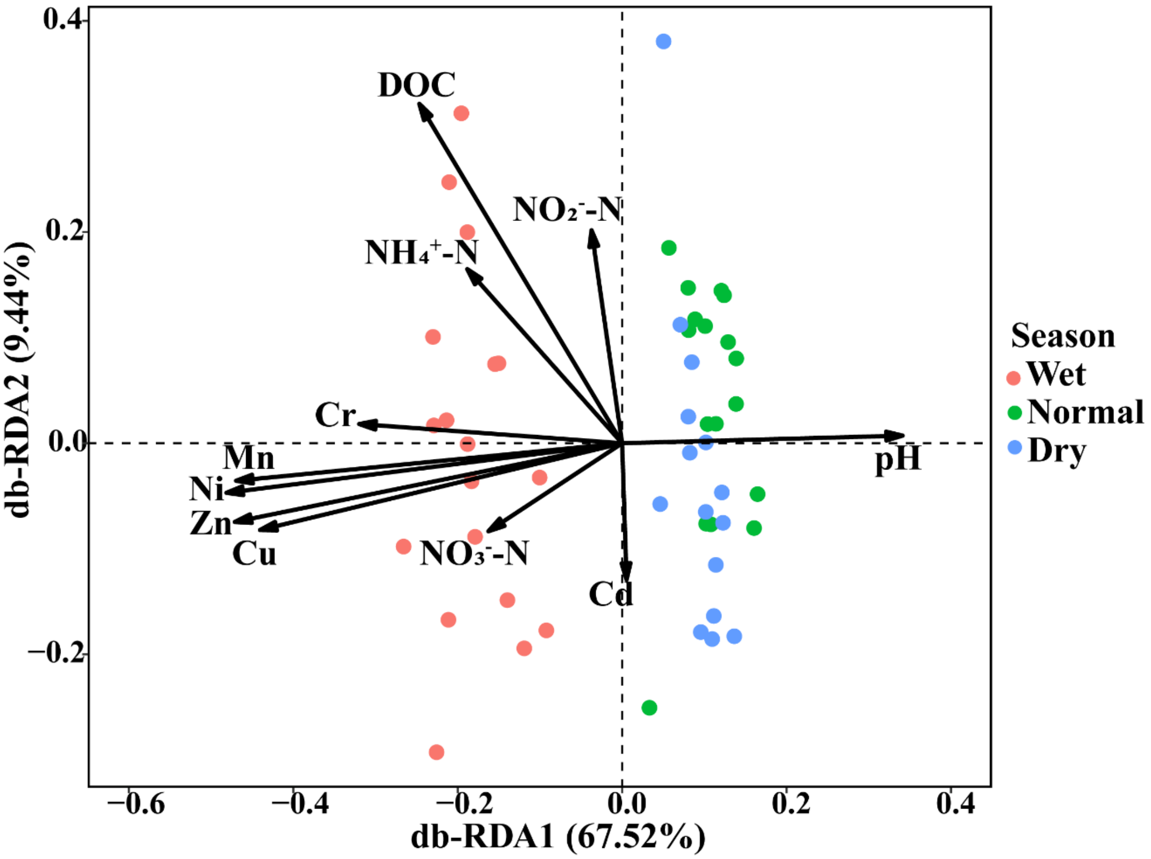

Figure S6 db-RDA showing the effects of environmental factors on DAMO bacterial communities.

Table S1 Samples collection of this study [1]

Sites	Wet	Normal	Dry	latitude (°N)	longitude (°W)
JH1	+	+	+	32.14	119.55
JH2	+	+	-	31.98	119.59
JH3	+	+	+	31.90	119.73
JH4	+	+	+	31.84	119.85
JH5	+	+	+	31.72	120.06
JH6	+	+	+	31.65	120.19
JH7	+	+	+	31.49	120.39
WY1	+	+	+	31.42	119.85
WY2	+	-	-	31.53	119.91
WY3	+	+	+	31.57	119.98
DJ1	+	+	+	31.56	119.46
DJ2	+	+	+	31.68	119.51
DJ3	+	+	-	31.83	119.60
YL1	+	+	+	31.38	119.78
YL2	+	+	+	31.39	119.65
YL3	+	+	+	31.40	119.57
YL4	+	+	+	31.40	119.53
YL5	+	+	+	31.44	119.32

[1] +: Sampling; -: No sampling

Table S2 Physicochemical attributes of each sample

Season	Sample	Cr (ug/g)	Mn (ug/g)	Ni (ug/g)	Cu (ug/g)	Zn (ug/g)	Cd (ug/g)	NH_4^+-N (ug/g)	NO_2^--N (ug/g)	NO_3^--N (ug/g)	DOC (ug/g)	pH
Wet	DJ1	71.708	811.163	27.921	32.119	132.369	0.436	26.331	0.038	1.677	82.288	7.49
	DJ2	66.830	815.074	26.734	36.228	115.688	0.439	22.016	0.030	3.473	111.840	7.68
	DJ3	71.716	699.290	27.485	32.036	107.750	0.425	33.409	0.024	2.516	84.807	7.43
	JH1	64.199	581.899	24.446	48.173	223.237	0.668	31.441	0.062	8.741	70.943	7.60
	JH2	65.564	733.466	24.525	23.589	119.100	0.359	32.672	0.048	4.589	108.826	7.57
	JH3	56.011	602.898	21.382	16.378	85.669	0.339	17.207	0.025	7.389	75.146	7.47
	JH4	56.810	444.504	23.572	15.426	90.895	0.297	17.746	0.084	1.633	146.387	7.66
	JH5	87.211	665.543	32.467	47.009	136.621	0.461	32.784	0.031	5.292	108.247	7.62
	JH6	86.480	528.606	32.164	29.583	119.522	0.318	15.309	0.037	2.137	123.228	7.68
	JH7	52.603	609.077	19.589	13.604	79.588	0.201	5.677	0.043	2.232	166.528	7.59
	WY1	64.217	728.088	28.971	37.660	156.364	0.366	24.291	0.013	3.901	64.453	7.65
	WY2	80.232	733.900	38.539	37.391	139.707	0.374	13.179	0.019	1.642	89.463	7.56
	WY3	79.105	460.857	31.379	31.772	106.501	0.254	26.939	0.016	1.378	51.337	7.56
	YL1	73.652	1065.632	34.312	43.048	139.999	1.132	53.256	0.051	10.142	105.535	7.41
	YL2	60.422	656.346	24.875	29.416	124.823	1.157	39.526	0.048	2.323	61.887	7.51
	YL3	54.464	491.661	20.768	19.851	105.192	0.935	16.255	0.030	3.282	50.942	7.28
	YL4	53.442	827.377	19.850	20.166	93.014	0.624	53.643	0.062	2.353	112.616	7.46

	YL5	55.144	761.920	21.461	14.265	64.324	0.417	14.402	0.031	2.987	54.219	7.48
Normal	DJ1	52.819	12.031	0.932	2.434	0.359	0.004	38.229	0.025	1.120	71.360	7.72
	DJ2	49.918	8.703	1.422	1.448	0.975	0.037	13.465	0.013	3.932	84.375	7.76
	DJ3	84.901	8.053	2.748	0.743	3.275	0.491	21.805	0.016	2.110	71.000	7.77
	JH1	45.370	10.077	1.012	2.623	0.428	0.002	11.789	0.024	6.581	116.261	7.79
	JH2	41.998	12.523	0.689	3.009	0.165	0.000	7.092	0.030	0.811	56.819	7.83
	JH3	39.627	12.887	0.623	3.201	0.149	0.000	5.518	0.033	0.962	55.169	7.81
	JH4	39.385	10.096	0.750	1.783	0.318	0.003	4.856	0.039	1.400	67.204	7.57
	JH5	58.429	8.017	1.926	1.333	1.416	0.047	34.588	0.027	0.270	74.012	7.72
	JH6	84.204	4.510	5.234	0.279	22.483	50.204	22.984	0.048	0.512	83.480	7.71
	JH7	55.596	6.955	1.716	0.570	1.984	0.184	27.529	0.068	0.063	86.020	7.82
	WY1	35.128	11.717	0.549	2.739	0.163	0.000	39.811	0.057	3.039	80.677	7.83
	WY3	74.706	6.363	3.686	0.553	6.821	1.914	2.947	0.068	0.654	50.289	7.70
	YL1	43.601	14.045	0.806	4.687	0.161	0.001	47.757	0.030	5.225	90.531	7.74
	YL2	40.241	10.557	0.733	2.061	0.260	0.005	19.609	0.089	2.841	71.883	7.68
	YL3	31.101	11.889	0.417	2.908	0.091	0.000	8.581	0.012	4.951	63.850	7.60
	YL4	33.540	13.991	0.424	4.316	0.067	0.000	10.626	0.046	5.356	62.482	7.73
	YL5	33.747	14.812	0.421	2.728	0.069	0.000	2.458	0.087	2.164	46.247	7.64
Dry	DJ1	44.356	12.661	0.768	3.148	0.275	0.001	50.855	0.053	0.026	131.273	7.81
	DJ2	52.318	9.290	1.476	1.902	0.756	0.011	43.556	0.056	0.550	78.397	7.84

JH1	34.055	13.082	0.437	4.646	0.079	0.000	2.344	0.057	1.595	57.562	7.80
JH3	34.502	13.578	0.471	3.360	0.090	0.000	3.256	0.062	0.533	37.463	7.80
JH4	47.183	8.634	1.559	1.626	0.800	0.096	3.934	0.007	0.250	77.526	7.79
JH5	47.458	16.838	0.731	4.702	0.107	0.000	11.689	0.055	1.635	44.806	7.83
JH6	77.798	6.084	4.057	0.479	10.328	5.575	12.772	0.015	0.213	61.966	7.86
JH7	28.898	13.537	0.281	3.152	0.035	0.000	1.826	0.009	0.289	46.249	7.78
WY1	58.626	10.853	1.589	1.385	0.831	0.015	9.170	0.024	0.025	43.115	7.71
WY3	62.694	7.997	2.139	0.901	2.060	0.109	3.033	0.009	1.111	36.779	7.85
YL1	51.307	12.769	1.112	4.032	0.254	0.001	17.985	0.039	0.362	78.607	7.86
YL2	31.033	13.695	0.349	4.942	0.042	0.000	13.556	0.012	1.319	79.318	7.87
YL3	46.162	8.680	1.306	1.740	0.811	0.072	12.406	0.028	1.296	77.907	7.86
YL4	31.836	11.462	0.416	2.921	0.088	0.000	5.148	0.012	1.524	55.136	7.85
YL5	20.164	15.537	0.137	6.248	0.007	0.000	2.352	0.013	1.451	49.404	7.78

Table S3 Identity alignment of representative sequence in each ASV with four typical DAMO bacterial genomes

	M. limnetica (%)	*M. oxyfera* (%)	*M. lanthanidiphila* (%)	*M. sinica* (%)
ASV_1	92.412	92.141	91.599	91.599
ASV_2	93.225	93.496	92.412	92.683
ASV_3	92.412	92.141	91.599	91.599
ASV_4	93.225	92.954	92.954	92.683
ASV_6	93.478	93.207	92.663	92.12
ASV_9	94.038	93.767	92.954	92.954
ASV_5	92.412	92.141	91.599	91.599
ASV_8	93.225	93.225	92.412	92.683
ASV_10	92.954	92.683	92.141	92.141
ASV_12	97.305	98.387	98.387	97.581

Table S4 Seasonal dynamics of subgroups of DAMO bacterial communities

Ratio of sequences (%)	Wet	Normal	Dry
Group A	4.49	63.80	31.71
Group B	31.13	37.12	31.75

Table S5 PERMANOVA test showing the differences of whole, abundant, and rare DAMO bacterial communities across different seasons

group	Whole		Abundant		Rare	
	F	*p*	F	*p*	F	*p*
All	10.674	0.001	14.099	0.001	1.071	0.001
Wet/Normal	18.26	0.001	25.627	0.001	1.101	0.001
Wet/Dry	11.183	0.001	15.646	0.001	1.079	0.001
Normal/Dry	1.75	0.061	1.777	0.072	1.031	0.268
